# In the era of humanitarian crisis, young women continue to die in childbirth in Mali

**DOI:** 10.1186/s13031-020-00334-5

**Published:** 2021-01-03

**Authors:** Pierre Coulibaly, Clémence Schantz, Brehima Traoré, Nanko S. Bagayoko, Abdoulaye Traoré, Fanny Chabrol, Oumar Guindo

**Affiliations:** 1 Hôpital Sominé Dolo de Mopti, RN6, Commune de Socoura, Sévaré, Mali; 2 CEMS-Centre d’Etude des Mouvements Sociaux; CNRS/EHESS FRE 2023 – INSERM U1276, Paris, France; 3 Centre Population et Développement (Ceped), Institut de recherche pour le développement (IRD) et Université de Paris, Inserm ERL 1244, 45 rue des Saints-Pères, 75006 Paris, France

## Abstract

Maternal mortality occurs mostly in contexts of poverty and health system collapse. Mali has a very high maternal mortality rate and this extremely high mortality rate is due in part to longstanding constraints in maternal health services. The central region has been particularly affected by the humanitarian crisis in recent years, and maternal health has been aggravated by the conflict. Sominé Dolo Hospital is located in Mopti, central region. In the last decade, a high number of pregnant or delivering women have died in this hospital.

We conducted a retrospective and exhaustive study of maternal deaths occurring in Mopti hospital. Between 2007 and 2019, 420 women died, with an average of 32 deaths per year. The years 2014–2015 and the last 2 years have been particularly deadly, with 40 and 50 deaths in 2018 and 2019, respectively. The main causes were hypertensive disorders/eclampsia and haemorrhage. 80% of these women’s deaths were preventable. Two major explanations result in these maternal deaths in Sominé Dolo’s hospital: first, a lack of accessible and safe blood, and second, the absence of a reference and evacuation referral system, all of which are aggravated by security issues in and around Mopti.

Access to quality hospital care is in dire need in the Mopti region. There is an urgent need for a safe blood collection system and free of charge for pregnant women. We also strongly recommend that the referral/evacuation system be reinvigorated, and that universal health coverage be strengthened.

## Background

Maternal mortality occurs mostly in contexts of poverty and health system collapse. In 2017, 295,000 women died in childbirth worldwide, 196,000 of them in sub-Saharan Africa [1]. Mali has a very high maternal mortality rate (562 women dying per 100,000 live births), and this extremely high mortality rate is due in part to longstanding constraints in maternal health services [1, 2]. Even though there has been progress worldwide, including in Africa, we would like to communicate the alarming figures of our retrospective study of maternal deaths in Sominé Dolo Hospital in the Mopti region over the course of a decade. Most of these deaths occurred among young women and could have been avoided if the health system had been strengthened and the region stabilised.

## The region of Mopti sinking into crisis

**Fig. 1:**
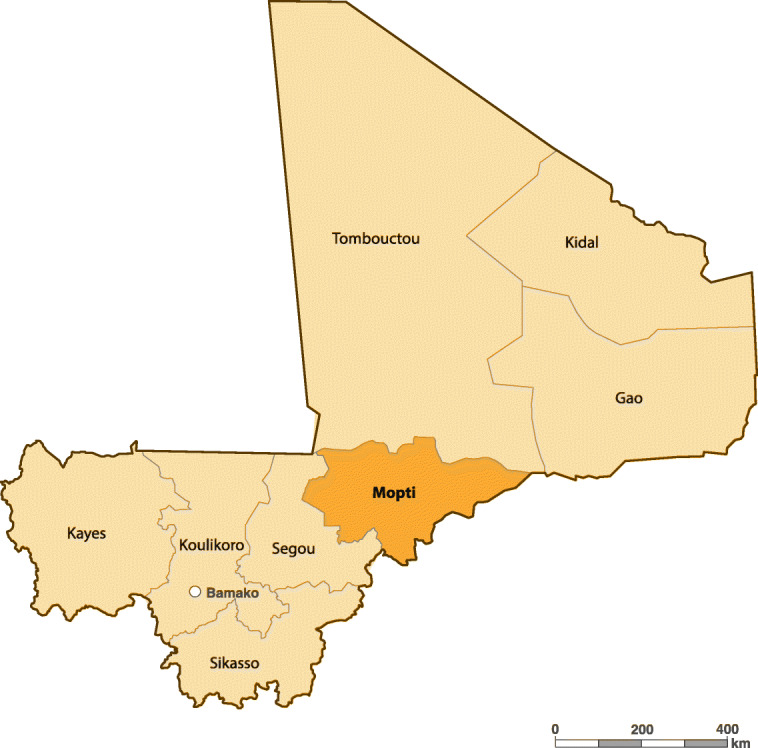
Map of Mali (source: IGM, E. Bonnet, IRD, 2020)

Sominé Dolo Hospital is located in central Mali, 675 km north of the capital city of Bamako (Fig. 1). The Mopti region has been particularly affected by the humanitarian crisis in recent years [3], and maternal health has been aggravated by the conflict [2]. The region of Mopti, once a tourist area, has been sinking into crisis for the last ten years. The region is agricultural and 46% of women are farmers [4]. The health system is organized in a pyramidal structure with community health centres at the bottom of the pyramid (first referral), reference health centres for second referral and then regional hospitals. The Sominé Dolo regional hospital in Mopti is therefore the last level of recourse in the region where women with a pathology are transferred (e.g. eclampsia, uterine ruptures, severe hemorrhages). In the Mopti region, during the last five years, 16% of women received no prenatal care during their pregnancy and 45.5% gave birth at home. 93% of women do not have medical insurance [4].

Sominé Dolo is the unique referral hospital for the region. The hospital moved within a new building in Sevare in 2012 but is still insufficiently equipped to face the increasing number of war injuries and inter-community casualties. To attend to pregnant women, the hospital has a gynaecological obstetrics department with 21 beds and qualified staff, including two gynaecologist obstetricians, six midwives and four obstetric nurses in 2019. In 2019, 1690 women gave birth in this hospital including 60% who were referred/evacuated from another health facility. In the last decade, a high number of pregnant or delivering women have died in this hospital.

## An exhaustive study of maternal deaths

We conducted a retrospective and exhaustive study of maternal deaths occurring in the service over the last 13 years, from 2007 to 2019. Maternal deaths were defined in this study as women who die during pregnancy or childbirth in the hospital. These figures are therefore underestimated compared to the official definition of maternal death, which includes women dying within 42 days postpartum. These data therefore do not include women who died at home or in other health centres in the region. The delivery registers, the maternal death register, the quarterly activity report from the hospital and the maternal death audit book constitute our main sources of data. The data were manually processed using a survey form that has been drawn up previously.

## Women arriving too late and dying too young

**Fig. 2:**
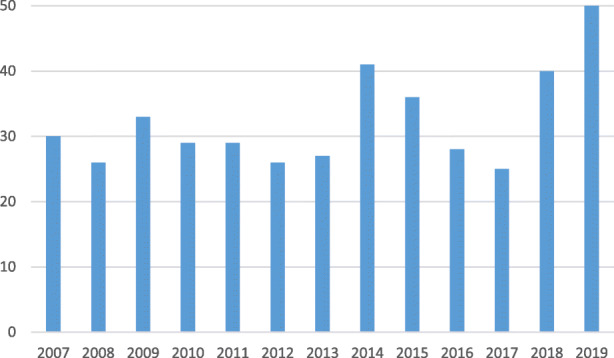
Number of maternal deaths between 2007 and 2019, Sominé Dolo Hospital, Mali

In Mopti hospital, 420 women died between 2007 and 2019, with an average of 32 deaths per year. The years 2014–2015 and the last 2 years have been particularly deadly, with 40 and 50 deaths in 2018 and 2019, respectively (Fig. 2). Forty percent of the women who died were young, between the ages of 18 and 25. The main causes were hypertensive disorders/eclampsia (26%) and haemorrhage (23%). More than half of these women (51%) died within the first hour of admission to the hospital, suggesting that they arrived in very serious condition. Of these 420 women who died, 164 cases could be audited. These audits showed that 80% of these women’s deaths were preventable. Tables 1 and 2 present women's trajectories in order to show the severity of the structural obstacles encountered by women and their families.

**Table 1:** Fanta’s story

Fanta arrives at Somine Dolo Hospital on January 30, 2018 at 2:00 am. She is 26 years old and pregnant for the fourth time with only one living child. She is brought by her parents by pirogue (15 km/h) for haemorrhage in the third trimester of pregnancy. She had stayed 14 h earlier in the community health centre of her locality in the north of the country in the Timbuktu region. This health centre is 200 km from the hospital in Mopti. We received her in a state of haemorrhagic shock due to a retroplacental hematoma. On admission the blood pressure and pulse are impregnable with cold extremities and conjunctival pallor. She has no donor, and there is no blood available in the laboratory compatible with her group. She died 15 min after admission in haemorrhagic shock without being transfused.

**Table 2:** Awa’s story

Awa is a 30 year old pregnant woman. This is her fifth pregnancy, she has three living children and an early neonatal death in her fourth pregnancy. She gave birth with great difficulty the last time. We receive her at the Sominé Dolo Hospital in Mopti on April 11, 2019 at 7:30 pm. She was evacuated from the health district of Bankass located 130 km from our hospital for uterine rupture. She did not make any prenatal visit. She has been in labor for 2 days at home. After the failed attempt to deliver at home, she was brought to the reference health centre in Bankass by animal-drawn cart. The diagnosis of uterine rupture was made and she arrived at the Sominé Dolo hospital by ambulance after a 3-h journey. She was very anemic, accompanied by her very elderly mother-in-law who was unable to give blood and weak. Her husband’s brother had to leave his village to come the next day. We performed a subtotal hysterectomy. She received a single bag of blood that belongs to another patient. She died 1 h after the operation. There were no other blood bag available for her.	

## Lack of blood and failure of the referral system

As these stories show, there are several explanations for the late recourse to care in Mali, including financial obstacles, but two other major explanations result in these maternal deaths in Sominé Dolo’s hospital: first, a lack of blood, and second, the absence of a reference and evacuation referral system. Mali has a serious shortage of blood for transfusion, and patients are often unofficially asked to pay for the blood they receive [5]. This failure of the reference and evacuation referral system has been aggravated by several crises in the Sahelian zone since 2012. Ambulances are targets of recurrent attacks, and patients experience hardship in accessing health centres, including a ban on travel from 6 p.m. in this region due to a lack of safety.

## Conclusion

Despite recent progress in reducing child mortality and improving maternal health in sub-Saharan Africa [6], facilities are still under-staffed and under-resourced in Mali [7] and require immediate action to eliminate preventable maternal deaths. The deaths of young women affect an entire family and community, and despite the user fee exemption policy related to caesarean section that was implemented in 2005 in Mali, families are still facing catastrophic expenditures related to emergency obstetric care. These high expenses (the highest of all being the costs of treatment) lead to serious long-lasting consequences that undermine the well-being of entire households, such as food insecurity, indebtedness and overall impoverishment [5]. With the COVID-19 pandemic, women’s sexual and reproductive rights in humanitarian and fragile settings are threatened more than ever [8] and low-income countries will face an increase in maternal deaths [9]. It is important to continue and systematise audit sessions for maternal deaths, as they are crucial in collectively documenting clinical cases and noting preventable maternal deaths [10]. We would like to call on health authorities to prioritise these lifesaving services. First, we recommand that the referral/evacuation system be reinvigorated and secured. Second we recommend that a blood bank be created to ensure the availability of blood products 24 h a day. Thirdly, and more broadly, quality of care and affordability should be comprised within a universal health coverage system.

## Data Availability

Data are available upon request to the corresponding author.
